# 3D Printing Properties of Heat-Induced Sodium Alginate–Whey Protein Isolate Edible Gel

**DOI:** 10.3390/gels10070425

**Published:** 2024-06-27

**Authors:** Zhihua Li, Siwen Wang, Zhou Qin, Wenbing Fang, Ziang Guo, Xiaobo Zou

**Affiliations:** School of Food and Biological Engineering, Jiangsu University, Zhenjiang 212013, China; 13361224526@163.com (S.W.); 19599960979@163.com (Z.Q.); 13952159113@163.com (W.F.); 2112018015@stmail.ujs.edu.cn (Z.G.)

**Keywords:** whey protein isolate, sodium alginate, 3D printing, rheological properties

## Abstract

The objective of this study was to develop a food 3D printing gel and investigate the effects of whey protein isolate (WPI), sodium alginate (SA), and water-bath heating time on the 3D printing performance of the gel. Initially, the influence of these three factors on the rheological properties of the gel was examined to determine the suitable formulation ranges for 3D printing. Subsequently, the formulation was optimized using response surface methodology, and texture analysis, scanning electron microscopy (SEM), and Fourier-transform infrared (FTIR) spectroscopy were conducted. The rheological results indicated that gels with WPI concentrations of 6–7 g, SA concentrations of 0.8–1.2 g, and water-bath heating times of 10–12 min exhibited lower yield stress and better self-supporting properties. The optimized formulation, determined through response surface methodology, consisted of 1.2 g SA, 6.5 g WPI, and a heating time of 12 min. This optimized formulation demonstrated enhanced extrusion capability and superior printing performance. SEM analysis revealed that the optimized gel possessed good mechanical strength, and FTIR spectroscopy confirmed the successful composite formation of the gel. Overall, the results indicate that the optimized gel formulation can be successfully printed and exhibits excellent 3D printing performance.

## 1. Introduction

3D printing, as a burgeoning additive manufacturing technology, employs a layer-by-layer deposition method to mold materials into a three-dimensional structure, utilizing various contemporary processing tools to achieve the desired configuration [1]. Currently, 3D printing technology finds applications in diverse industries, such as construction [2], aerospace [3], and the medical field [4]. In the food industry, 3D printing is gaining significant attention due to its ability to create customized and intricate food products with precise control over the ingredients and shapes. Presently, an array of materials is available for food 3D printing, including dough [5], surimi [6], fruits [7], protein [8], and more. Among these, extrusion 3D printing stands out as the most widely adopted technology for food manufacturing. It relies on extrusion and layer-by-layer deposition to create a three-dimensional structure [9]. In extrusion 3D printing, the characteristics of the food material play a pivotal role. Smooth extrusion and the ability to maintain stability are crucial attributes for the ink used in extrusion 3D printing. Consequently, materials with shear-thinning properties, such as gels, are deemed suitable for the 3D printing of food [10].

Whey protein isolate (WPI) is a proteinaceous substance derived from whey, encompassing all essential amino acids crucial for maintaining human health, rendering it nutritionally valuable. It is extensively utilized as a by-product in the dairy industry due to its widespread availability, nutritional richness, cost-effectiveness, and favorable biocompatibility [11]. The global market for whey protein is projected to reach USD 14.5 billion by 2026, highlighting its significant economic impact. Given the robust emulsification and gelation capabilities of whey protein isolates, researchers commonly employ heat treatment to prepare WPI-based gel systems [12]. Elevated temperatures induce alterations in the spherical structure of WPI, exposing hydrophobic groups. Through the cross-linking effect between protein molecules and the formation of covalent disulfide bonds, a three-dimensional mesh structure of proteins emerges, culminating in gel formation. This covalent cross-linking imparts robust mechanical and structural stability to the gel material [13].

Sodium alginate, a prevalent hydrocolloid, is frequently employed in hydrogel formation due to its heightened stability to heat, acid, and alkali, coupled with high biocompatibility [14]. In the field of food, sodium alginate is widely used to improve food processing, taste, and texture characteristics because of its good safety and its edible nature [15]. The market value of sodium alginate was estimated at USD 760 million in 2023, and it is expected to grow steadily, reflecting its extensive application in the food industry. When sodium alginate and whey protein isolate aqueous solution were heated together, sodium alginate was dispersed in the three-dimensional grid structure formed by whey protein isolate as a filler, which improved the strength of the gel. An appropriate addition of sodium alginate also enhances the rheological properties of whey protein isolate gels [16]. In response to the aforementioned considerations, this study endeavors to develop a hydrogel for 3D printing with sodium alginate as a filler and whey protein isolate as the primary component. This innovative hydrogel targets fitness and athlete populations with a demand for high protein consumption. The investigation mainly studied three pivotal factors—the addition of amounts of sodium alginate and the addition of amounts of whey protein isolate, along with water-bath heating time—evaluating their impact on printing behavior. Utilizing response surface optimization, the study aims to derive the most suitable gel formulation for 3D printing. The unique properties of WPI and SA, such as their nutritional benefits and gel-forming capabilities, make them ideal candidates for developing a stable and printable food gel. By optimizing the formulation and processing conditions, this research seeks to enhance the mechanical and rheological properties of the gel, ensuring its suitability for 3D printing. This study not only contributes to the advancement of food 3D printing technology but also opens new avenues for creating personalized food products for consumers.

## 2. Results and Discussion

### 2.1. Rheological Properties of Gels

**Figure 1:**
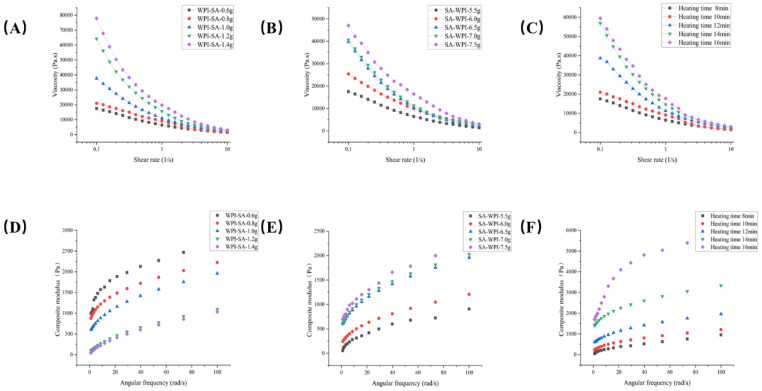
(**A**) Effect of sodium alginate on apparent gel viscosity. (**B**) Effect of WPI on apparent gel viscosity. (**C**) Effect of heating duration on apparent gel viscosity. (**D**) Effect of sodium alginate on gel complex modulus. (**E**) Effect of WPI on gel complex modulus. (**F**) Effect of heating duration on gel complex modulus.

The rheological behavior of food 3D printing inks plays a crucial role in predicting ink behavior and ensuring the quality of the printed product. The investigation involves analyzing data obtained after conducting rheological tests on the gel in a one-way experiment, with a primary focus on the extrusion stage’s influence on printing behavior. Key parameters such as the apparent viscosity of the ink and the yield stress during extrusion significantly impact the ease of extrusion. Figure 1A–C illustrates the impact of sodium alginate addition, WPI addition, and water-bath duration on the apparent viscosity of the gel. The figure reveals a notable increase in the apparent viscosity with higher concentrations of sodium alginate. Similarly, increased WPI addition and prolonged water-bath duration result in the elevated apparent viscosity. Moreover, all gels exhibit a decrease in viscosity with an increase in shear rate, indicative of shear-thinning behavior [17]. During the heating of the water bath, the WPI is denatalized, the protein structure is destroyed, and the hydrophilic and hydrophobic groups are exposed, resulting in the increased intermolecular forces of the protein, resulting in the formation of a network structure of the protein, which significantly increases the viscosity of the solution. During the heating of the water bath, the molecular chains of sodium alginate expand and become more fully cross-linked, thus making the gel network tighter, which also leads to an increase in the viscosity of the gel. The viscosity curves, when fitted, consistently yield flow coefficients (n) less than 1, effectively confirming the shear-thinning nature of the emulsions [18]. The coefficient of consistency (K) influences the content of hydrophilic groups in the gels, thereby affecting gel consistency (Table 1).

During the forming stage, the complex modulus serves as a key indicator of the material’s ability to maintain stability and mechanical strength. Figure 1D–F depicts the self-supporting strength of the gel under varying conditions. When the amount of WPI is insufficient and the heating time is shorter, the self-supporting strength of the gel diminishes. This inadequacy results in challenges during the formation of the gel after the printing process. When the amount of sodium alginate is insufficient, there is a lack of filler in the gel network formed by WPI. This deficiency leads to insufficient gel consistency and a notable reduction in self-supporting capability. The presented data underscore the critical role of proper formulation, particularly in terms of WPI and sodium alginate quantities, to achieve optimal self-supporting strength during the forming stage of 3D printing [19].

The results from amplitude scanning reveal a correlation between the addition of sodium alginate, WPI, and increased heating time with a corresponding rise in the gel’s yield stress. This phenomenon can be attributed to the influence of WPI and sodium alginate on elevating gel consistency. The use of the power law equation on the viscosity curve supports this observation, and the high values of R^2^ (greater than 0.99) indicate the accurate representation of the gel’s viscosity profile by the power law equation.

Based on the rheological results, the following conditions yield a gel with favorable properties, including lower yield stress, improved self-supporting characteristics, and a reasonable apparent viscosity range: Sodium Alginate Addition: 0.8–1.2 g, WPI Addition: 6–7 g, Heating Time: 10–12 min. These specific ranges of sodium alginate addition, WPI addition, and heating time collectively contribute to the desired rheological properties of the gel. Achieving lower yield stress enhances extrudability, while the improved self-supporting properties indicate suitability for 3D printing. Gels with all factors in the above range have better 3D printing performance.

### 2.2. Parameter Optimization

**Figure 2:**
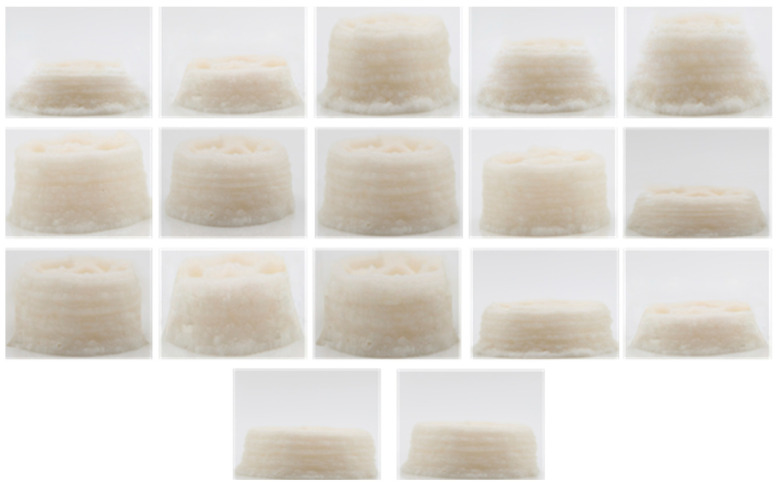
Responsive surface design recipe printable images.

Using the second-order response surface regression model and taking the comprehensive score of the printed product in Figure 2 as the response variable, regression fitting analysis was conducted on the above three factors, and the regression equation of the comprehensive score was obtained as follows:

Y (composite score) = −1076.87 − 856.275 ∗ A + 475.452 ∗ B + 20.4981 ∗ C + 87.175 ∗ AB +7.23125 ∗ AC + 11.94 ∗ BC + 143.875 ∗ A^2^ − 56.15 ∗ B^2^ − 4.76063 ∗ C^2^

Analysis of variance (ANOVA) of the response surface model regression equations revealed that the primary terms A, B, and C as well as the interaction terms AB and BC and the secondary terms A^2^, B^2^, and C^2^ had highly significant effects on the composite scores, while the interaction term AC had insignificant effect on the composite scores. The order of factors affecting the composite scores of the samples was obtained as C > A > B, i.e., C water-bath heating time > A sodium alginate addition > B whey protein isolate addition. The regression model established with F = 108.55, *p* < 0.01, indicated that the significance of the regression model reached a highly significant level (*p* < 0.01); meanwhile, the *p* value of the misfit term was 0.0514 > 0.05, and the difference of its model was not significant, which indicated that the non-experimental factors have less influence on the composite score, and the model has good experimental stability and is less disturbed by the non-experimental factors, which indicates that the equation is reliable. The regression coefficient *R*^2^ is 99.29% > 85%, indicating that the test model fits well with the actual test, and about 99.29% of the results of the actual test can be interpreted and analyzed by the fitted model, so the regression equation can be used to replace the true factor values of the test to analyze the correspondence between the values of each factor and the composite score [20]. The calibration coefficient *R*^2^*_Adj_* is 0.9837, which is basically close to *R*^2^, proving that the model is sufficiently accurate and generalizable as well as reasonable. The prediction *R*^2^*_Pre_* is 0.9037, and the difference between the prediction *R^2^* and the correction coefficient *R*^2^*_Adj_* is less than 0.2, indicating that the prediction results are reliable. The specific analysis results are detailed in Table 2.

In order to assess the model’s reliability accurately, two metrics were employed: the standardized coefficient of variation (C.V. %) and the signal-to-noise ratio (Adeq Precision). A C.V. % below 10 is generally indicative of good repeatability, and a signal-to-noise ratio above 4.0 signifies a robust fit of the model to the test values. As per the test results in Table 2, the C.V. % of the regression model is 5.09, and the Adeq Precision is 29.1717, both meeting the criteria for reliability [21].

**Figure 3:**
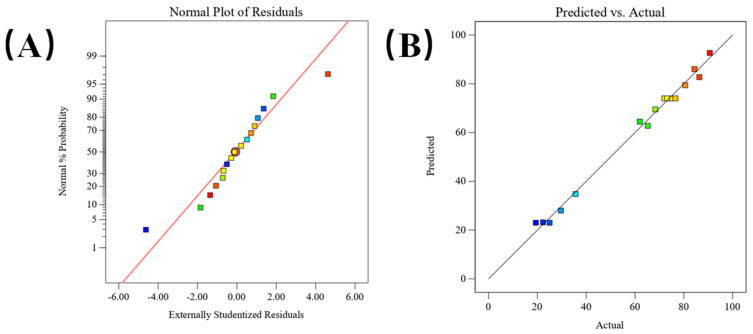
(**A**): Figure of normal plot of residuals; (**B**): Figure of predicted vs. actual.

The evaluation of T-ization residuals involves representing data points through a scatter plot to assess linearity and even distribution around a line. Figure 3 illustrates this analysis, showcasing linear dispersion, and T-chemical residuals conforming to a normal distribution [22]. Additionally, Figure 3 presents a distribution plot of actual versus predicted values. The concentration of data points around a straight line indicates the proximity of actual values to predicted values, affirming the validity of the predictive regression model.

**Figure 4:**
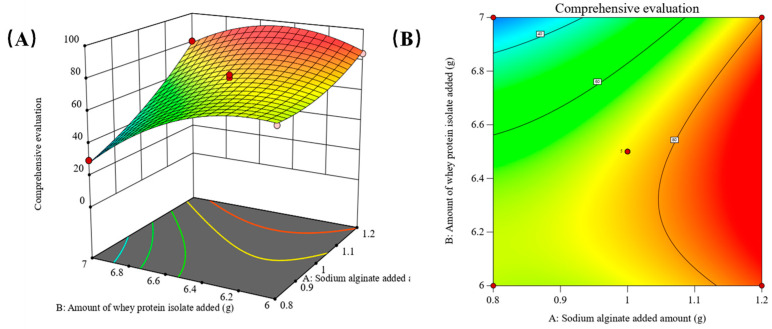
(**A**) The three-dimensional plot (**B**) The contour plot showing the interaction between A the sodium alginate added amount and B the amount of whey protein isolate added on the response.

**Figure 5:**
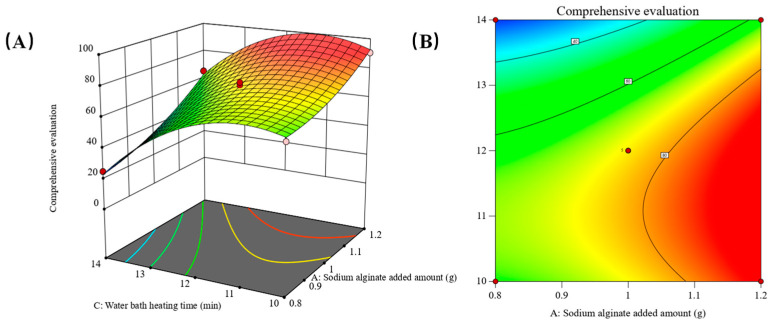
(**A**) The three-dimensional plot (**B**) The contour plot showing the interaction between A the sodium alginate added amount and C the water-bath heating time on the response.

**Figure 6:**
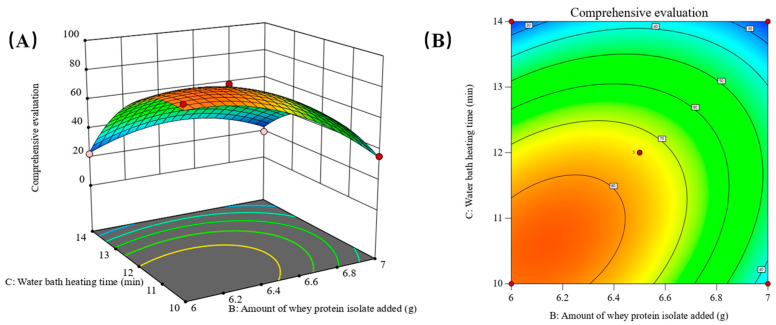
(**A**) The three-dimensional plot (**B**) The contour plot showing the interaction between B the amount of whey protein isolate added and C the water-bath heating time on the response.

Building upon the ANOVA results of the regression model, Design-Expert 13.0.6 software was employed to generate response surface plots and contour plots. These plots are derived from the ANOVA results of the regression equation model. By fixing one factor at the intermediate level, the contour plots and response surface plots illustrate the impact of the interaction between the other two factors on the composite score. The outcomes of this analysis are visually presented in Figure 4, Figure 5 and Figure 6.

In Figure 4, with the heating time of Factor C (water bath) held constant at the intermediate level, the behavior of the composite score varies with the levels of sodium alginate (Factor A) and whey protein isolate (Factor B). Specifically, at low levels of whey protein isolate (B) addition, the composite score stabilizes with increasing sodium alginate (A) until a certain point, after which it rises rapidly. Conversely, at high levels of whey protein isolate addition, the composite score shows a more pronounced increase with rising sodium alginate levels. The change trend of the composite score is not consistent when whey protein isolate is added at low or high levels. Similarly, when sodium alginate is added at lower and higher levels, the change trend of the composite score varies with the increase in whey protein isolate. The contour plots further emphasize the significant interaction between sodium alginate and whey protein isolate. Notably, when sodium alginate is added at 1.1–1.2 g and whey protein isolate is added at 6.1–6.7 g, the predicted composite score is in a higher range. The *p* (AB) < 0.01 result and the steeper slope of the 3D plot affirm the substantial impact of the interaction between sodium alginate and whey protein isolate on the composite score.

As shown in Figure 5, when the factor B whey protein isolate addition was constant at the intermediate level, the composite score first increased and then decreased with the increase of C water-bath heating time, and the composite score gradually increased with the increase of A sodium alginate addition. Contour plot contour lines are more sparse; with the ANOVA results showing *p* (AC) > 0.05, the interaction between the two factors is not significant and the effect on the results is small [23].

As shown in Figure 6, when the factor A sodium alginate addition is at the intermediate level and unchanged, and when the B whey protein isolate addition is at the low level, the composite score gradually decreases with the increase in the C water-bath heating time; when the B whey protein isolate addition is at the high level, the composite score increases and then decreases with the increase in the C water-bath heating time; when the C water-bath heating time is at the low and high levels, the composite score changes with the increase in the B whey protein isolate addition. When the C water-bath heating time is at low and high levels, with the increase in B whey protein addition, the trend of change showed significantly different situations, respectively, declining or being elevated after the decline of different changes in the gauge, which can show that the interaction between the two factors is strong, the effect on the results is significant, the response surface of the 3D plot is steeper, and the results of the variance with *p* (BC) < 0.01, reached the level of highly significant. From the contour plots, it can be seen that the results were better when the B whey protein isolate addition was in the range of 6 to 6.3 g and the C water-bath heating time was in the range of 10 to 11 min.

In summary, the interaction terms AB and BC all reached highly significant levels of influence on the composite score of the test results, and the *p*-values of the variance results were less than 0.01, which is a significant interaction and has a greater degree of influence on the composite score of the results. The interaction term AC has a smaller degree of influence [24]. From the above data and from analyzing the charts, it can be seen that the order of influence of the two-factor interaction term on the composite score of the samples is BC > AB > AC.

**Figure 7:**
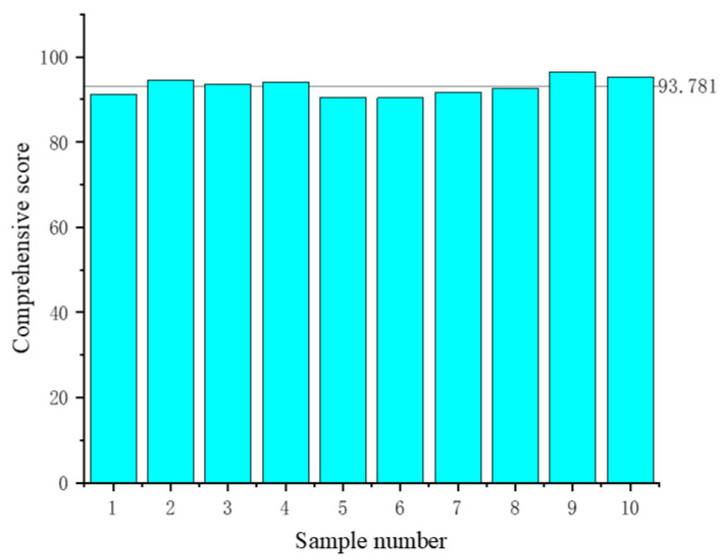
Comparison of the comprehensive score of the newly printed sample with the predicted comprehensive score.

**Figure 8:**
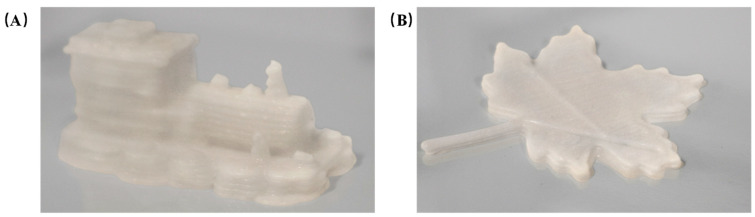
(**A**,**B**) Printing results of optimized formulations.

In order to optimize the effect of the comprehensive score, the conditions were optimized by using the effective regression model, and the best parameters were obtained as follows: sodium alginate addition: 1.199 g, whey protein isolate addition: 6.515 g, water-bath heating time: 12.308 min; and the comprehensive score was predicted to be 93.781. Considering the practicability and rationality of the experimental conditions, the optimized formula was 1.2 g of isolated whey protein, 6.5 g of isolated whey protein, and 12 min of water-bath heating time. In order to verify the effectiveness of the model and the printing performance of the optimized formula, the hollow cylinder model was printed again and the comprehensive score was calculated. The comprehensive score of the newly printed ten models was compared. As can be seen in Figure 7, the comprehensive score of the optimized formula was close to the predicted score of the response surface model, which confirmed the effectiveness of the regression model. The significant increase in the comprehensive score confirmed the improvement of the printing performance of the optimized gel formula. The optimized formula was printed for other models, and the printing effect was shown in Figure 8. As can be seen from the figure, the effective height of printing was effectively improved, and the deformation was significantly reduced.

### 2.3. Textural Properties

**Figure 9:**
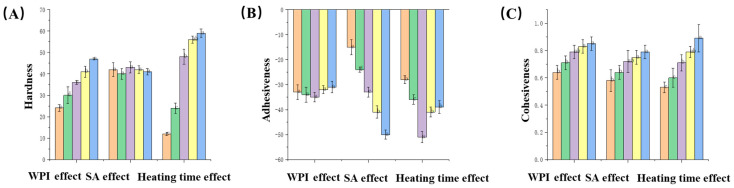
(**A**) The influence of three factors on the hardness of the gel; (**B**) The influence of three factors on the gel viscosity; (**C**) The influence of three factors on gel cohesiveness.

Texture analysis is a sensory manifestation of food materials and structural properties, and an important factor for consumers’ preference and acceptability of food. Figure 9 shows the test results of texture properties of gels in the single-factor experiment. It can be seen from the figure that the hardness of the gels increases significantly when the amount of WPI is increased, which is due to the interaction of protein molecules in the gels, forming cross-linking and aggregation [25]. A dense gel network was formed, and the mesh density increased when the WPI content increased, resulting in an increase in gel hardness. Meanwhile, the addition of SA had no obvious effect on gel hardness; and the gel hardness also increased as the heating time became longer, which was because the intramolecular covalent bond was broken when the protein was heated, and the hydrophobic effect between molecules became stronger. Causing molecules to attract each other to form clusters or gels, this new structure increases the interaction between molecules, making them harder. With the increase in WPI content, the cohesiveness of the gel will also increase; WPI plays a role in the formation and stability of the gel structure, so the increase in WPI content makes the gel structure more stable and compact. With the increase in SA content, the cohesion will also be significantly improved, which is because polysaccharide molecules have many hydrogen bonding sites [26]. These sites can interact with water molecules to form a cross-linked network to enhance the cohesion of the gel. With the increase in heating time, the hydrogen bonds inside the gel are destroyed, resulting in more free groups being exposed, and the interaction of free groups is enhanced, thus increasing the adhesion between molecules and enhancing the cohesion. According to the results of texture analysis, the addition of whey protein isolate has no significant effect on the viscosity of the gel, while the addition of sodium alginate makes the gel network more compact and complex, resulting in an increase in viscosity. In the initial process of water-bath heating, the interaction between protein molecules is enhanced to form aggregates, and the combination with sodium alginate makes the gel structure more stable, resulting in an increase in viscosity, and as the water-bath heating time further increases, it will lead to the replacement of protein aggregates, making the tight gel network structure loose, and ultimately resulting in a decrease in viscosity.

### 2.4. SEM Analysis

**Figure 10:**
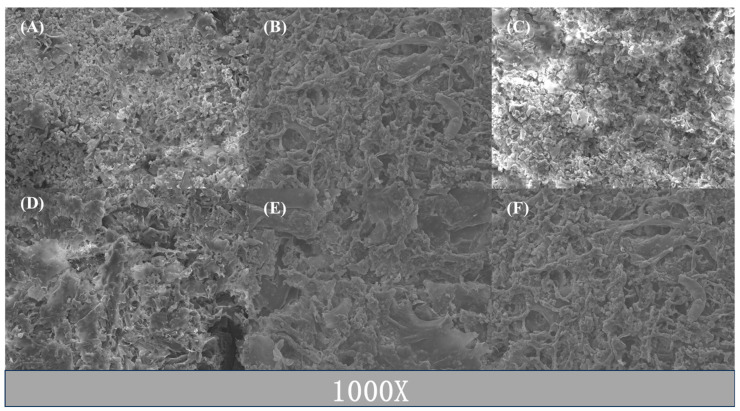
(**A**–**C**) Effect of WPI on the microstructure of gel. (**D**,**E**) Effect of SA on the microstructure of gel. (**F**) Effect of heat treatment on gel structure.

As the WPI content increased from 5.5 g to 6.5 g, the gel exhibited a progressively uniform structure, with sodium alginate (SA) uniformly diffusing throughout the gel network, as depicted in Figure 10A,B. However, with a further increase in WPI content, the protein formed larger aggregates, disrupting the uniform distribution of SA and occupying a more extensive space. Figure 10C illustrates that the addition of SA, with a higher polysaccharide concentration, led to broader WPI associations, enhancing the incompatibility between WPI and SA and resulting in the formation of a more stable porous structure, as observed in Figure 10D,F. Heat treatment expanded the protein structure, exposing hydrophobic groups [27]. The microstructure in Figure 10F demonstrates the increased spatial expansion and aggregation of proteins under the influence of 12 min heat treatment.

### 2.5. FTIR Analysis

**Figure 11:**
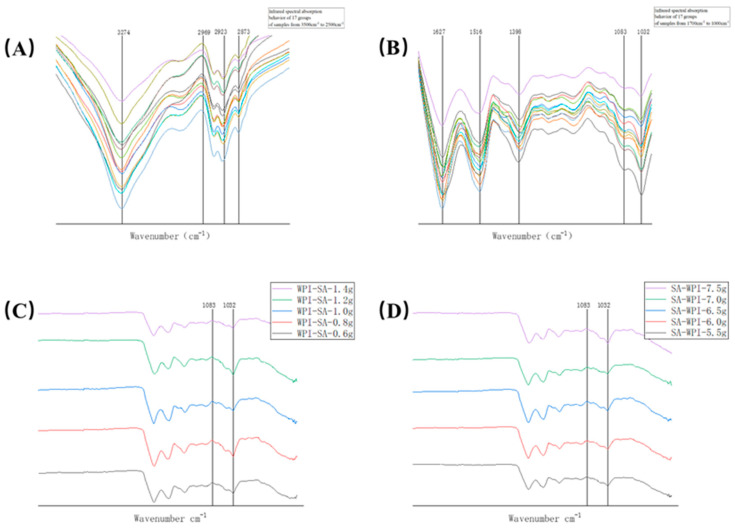
(**A**,**B**) Infrared spectral absorption behavior of all samples in response surface experiments. (**C**) Infrared spectral absorption behavior of samples with different sodium alginate additions (0.6–1.4 g). (**D**) Infrared spectral absorption behavior of samples with different whey protein isolate additions (5.5–7.5 g).

The surface functional groups of the experimental material were analyzed and characterized using FTIR, the characterization results are shown in Figure 11. The infrared spectrum of 17 groups of samples exhibited similarity, with the hydroxyl group’s stretching vibration peak observed at 3274 cm^−1^. Absorption peaks at 2969 cm^−1^, 2923 cm^−1^, and 2873 cm^−1^ originated from the stretching vibration of C-H bonds in -CH3- and -CH2-. The characteristic peak of C-C rigid vibration appeared at 1980 cm^−1^. Additionally, wavelengths 1627 cm^−1^ and 1516 cm^−1^ represented amide I bands, corresponding to the expansion of peptide bonds C=O in proteins, while 1536 cm^−1^ indicated amide II bands, mainly associated with the C-N expansion and N-H bending patterns of peptide bonds in proteins [28]. The asymmetric and symmetrical stretching vibrations of the carboxylate group in sodium alginate were observed at 1516 cm^−1^ and 1398 cm^−1^, respectively, leading to an overlap with the amide I band in the infrared spectrum. Furthermore, the C-O bond stretching vibration of the alcohol hydroxyl group in sodium alginate was noted at 1083 cm^−1^ and 1032 cm^−1^. These findings confirm the successful combination of WPI and SA.

When comparing the effects of varying sodium alginate and WPI contents on the functional groups within the gel, the increased content of sodium alginate and WPI resulted in stronger peak intensities of the amide I and amide II bands at 1083 cm^−1^ and 1032 cm^−1^. The secondary structure of WPI primarily relied on the hydrogen bond formation between the C=O bond in the skin chain and the N-H bond on the amide bond. The heightened intensity of the amide bond indicated a tighter binding of polysaccharides to WPI, suggesting no alteration in the secondary structure of whey protein isolates. Changing the reaction conditions only impacted the intensity of the amide I and amide II bands without any displacement, signifying that the secondary structure of whey protein isolates remained unchanged. Additionally, the addition of sodium alginate did not influence the conformation of the polypeptide chain.

## 3. Conclusions

Through the optimization of various parameters, a gel formulation suitable for 3D printing was successfully obtained, facilitating the realization of 3D printing with whey protein isolate. The printing results showed that after optimization by second-order response surface regression model, when 6.5 g whey protein isolate and 1.2 g sodium alginate were added and heated in a water bath for 12 min, the gel had an enhanced extrusion capacity and an excellent print reduction rate. The results showed that the addition of WPI and SA significantly affected the rheological properties and texture properties of the gel. The optimized formulation results in a uniform and dense microstructure. In summary, this study provides a new method for the production of protein-based foods and a new perspective for promoting the application of 3D printing in protein-based foods.

## 4. Materials and Methods

### 4.1. Materials

Whey protein isolate (WPI), acquired from Hilmar Ingredients Ltd. (Hilmar, CA, USA), exhibited a composition comprising 93% protein on a dry basis, with 4.7% moisture and 2.7% ash, as provided by the manufacturer; sodium alginate (SA), purchased from Shanghai Maclin Biochemical Technology Co., Ltd. (Shanghai, China), was derived from brown algae.

### 4.2. Preparation of WPI–SA Gel for 3D Printing

In this study, various quantities of sodium alginate (0.6, 0.8, 1.0, 1.2, and 1.4 g) were dissolved in 50 mL of deionized water [29]. The mixture was subjected to simultaneous rotation, stirring, and water-bath heating to ensure complete dissolution, resulting in aqueous sodium alginate solutions. Different amounts of whey protein isolate (WPI) (5.5, 6.0, 6.5, 7.0, and 7.5 g) were then incorporated into these solutions, forming aqueous gels [30]. These gels were subsequently heated in a water bath at 100 °C for varying durations (8, 10, 12, 14, and 16 min). After heating, the prepared hydrogels were stored at 4 °C for 12 h before being used in the printing process.

### 4.3. Model Design

**Figure 12:**
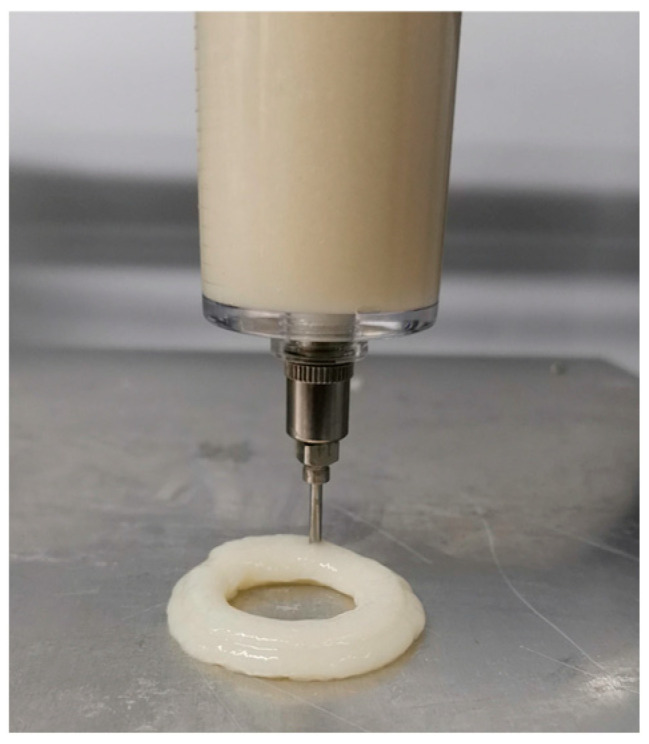
Printing process of the hollow cylinder model.

Using SolidWorks, a hollow cylinder was designed as the printing model. The diameter of the hollow cylinder was 50 mm, the thickness was 3 mm, and the height was 40 mm; the internal filling degree was specified as 20% to ascertain the printing reduction. Import the model established by Solidworks into slicing software (Cura 15.02.1) for slicing, and convert it into an STL format file, and apply it to printing, the printing process is shown in Figure 12.

### 4.4. Rheology Characterization

A rotational rheometer (HR-1, TA, New Castle, DE, USA) configured with a parallel plate featuring a 40 mm diameter and a 1 mm gap was employed to assess the gels [31]. The samples were positioned on the sample stage for 1 min to equilibrate to the measurement temperature (25 °C) and subsequently scraped to eliminate any excess gel that could impact the results. The conducted tests encompassed frequency scanning in oscillatory mode within the range of 1–100 rad/s (strain: 0.1%), amplitude scanning in oscillatory mode (amplitude: 0.01–100%), and viscosity profiling at varied shear rates (shear rate: 0.1–10 s^−1^) [32].

### 4.5. Parameter Optimization

#### 4.5.1. Determination of the Range of Independent Variables through One-Way Experiments

The range of independent variables underwent determination and optimization through one-way experiments. Key parameters influencing printing behavior were identified as the amount of sodium alginate added (0.6, 0.8, 1.0, 1.2, 1.4 g), the amount of WPI added (5.5, 6.0, 6.5, 7.0, 7.5 g), and the duration of water-bath heating (8, 10, 12, 14, and 16 min). Other parameters were fixed, with the sodium alginate addition set at (0.6, 0.8, 1.0, 1.2, 1.4 g), water-bath heating time at (8, 10, 12, 14, and 16 min), and the remaining parameters standardized to a sodium alginate addition of 1.0 g, a WPI addition of 6.5 g, and a water-bath heating duration of 12 min.

#### 4.5.2. Comprehensive Score of Printing Reduction Degree

The effective molding height and lateral deformation angle of the printed product are usually used to evaluate the quality of the printed product, expressed as follows:

$$y=(\frac{h}{30}\times k_{1}+\frac{180-\alpha }{180}\times k_{2})\times 100$$

Here, *h* represents the effective height of the printed model (unit: mm), *α* is the side bending angle of the model, and 100 is the unit coefficient. The coefficients *k*_1_ and *k*_2_ account for the proportionate weight of the effective height and side bending angle in the score. Based on pre-experimental observations, adopting values of *k*_1_ = 0.8 and *k*_2_ = 0.2 better reflects the impact of the print product.

#### 4.5.3. Response Surface Method

Using Design-Expert (13.0.6, USA), a three-factor, three-level Box–Behnken Design (BBD) was implemented, experimental factors and levels are shown in Table 3. The factors considered were sodium alginate addition, WPI addition, and heating time in the experiments. The response variables were the effective height of the printed product and the side bending angle [33]. The maximum, minimum, and central point values in the response surface experiment (coded −1, 1, 0, respectively) were determined by the results of the rheological characterization. To prevent the influence of confounding factors on experimental results, the tests were conducted in a randomized manner [34].

### 4.6. Textural Analysis of Gels

The textural properties of the samples were assessed using a texture analyzer (TA XT PLUS 1101927S, Stable Micro Systems, England, UK). The analyzer was calibrated with a 1 kg weight and equipped with a flat-ended aluminum probe having a diameter of 25 mm. The analysis employed the TPA (Texture Profile Analysis) measurement mode, incorporating a pre-test speed of 5 mm/s, an intra-test speed of 1 mm/s, and a post-test speed of 5 mm/s. All measurements were conducted at room temperature (25 ± 1 °C), and each test was repeated three times for robustness [35].

### 4.7. Scanning Electron Microscope (SEM)

A Scanning Electron Microscope (SEM) model JSM-7001F from Tokyo, Japan was employed for the observation and analysis of the microstructures of the lyophilized gels. The solid powders derived from the gels with various formulations were gold-sprayed and subsequently placed under vacuum conditions to obtain detailed microstructural information of the targets at a magnification of 1000× [36].

### 4.8. Fourier Infrared Spectroscopy

The sample, freeze-dried and ground into powder, was tested by Thermo Co Scientific Nicolet iS50 (Procured from Fremont, CA, USA), and the spectral scanning range was 4000–400 cm^−1^ with a resolution of 2 cm^−1^, using air as a reference, to analyze the secondary structure of the gel.

### 4.9. Statistical Analysis

All experiments were conducted three times. The mean value, error, and significance analysis of the results were reported. The Duncan multi-range test was used to conduct significance analysis of the data, and Origin 2019 was used to plot the results.

## Figures and Tables

**Table 1:** Yield stress (τ), consistency coefficient (K), flow coefficient (*n*), and fitting exponent (R^2^) for different gels in a one-way experiment.

	SA					WPI					Heating Time				
	0.6–1.4 g					5.5–7.5 g					8–16 min				
τ (Pa)	25.75	44.13	65.74	127.91	197.55	35.26	57.22	65.74	139.37	192.34	49.660	55.340	65.740	141.671	157.340
K (Pa∗S^n^)	88.0 ± 1.7	93.6 ± 1.4	110.1 ± 1.5	151.9 ± 2.1	187.8 ± 0.00	97.3 ± 1.3	101.3 ± 0.5	115.9 ± 2.5	154.5 ± 3.0	201.3 ± 3.7	59.200 ± 1.500	63.5 ± 1.6	113.400 ± 2.000	148.4 ± 3.2	204.5 ± 3.2
*n*	0.367 ± 0.007	0.450 ± 0.008	0.530 ± 0.013	0.593 ± 0.011	0.381 ± 0.006	0.447 ± 0.012	0.504 ± 0.011	0.567 ± 0.008	0.412 ± 0.008	0.430 ± 0.010	0.396 ± 0.008	0.379 ± 0.006	0.427 ± 0.010	0.425 ± 0.018	0.376 ± 0.004
R^2^	0.99	1	1	1	0.99	0.99	1	0.99	1	0.99	0.99	1	0.99	0.99	0.99

**Table 2:** Variance analysis for the established regression model.

Source of Variance	Square Sum	Degrees of Freedom	Mean Square	F	*p*	Significance
Model	9460.13	9	1051.13	108.55	<0.0001	**
A	2305.88	1	2305.88	238.14	<0.0001	**
B	1156.08	1	1156.08	119.39	<0.0001	**
C	2543.63	1	2543.63	262.69	<0.0001	**
AB	303.98	1	303.98	31.39	0.0008	**
AC	33.47	1	33.47	3.46	0.1054	
BC	570.25	1	570.25	58.89	0.0001	**
A^2^	139.45	1	139.45	14.40	0.0068	**
B^2^	829.69	1	829.69	85.69	<0.0001	**
C^2^	1526.81	1	1526.81	157.68	<0.0001	**
Residual	67.78	7	9.68			
Lost proposal	56.21	3	18.74	6.48	0.0514	
Pure error	11.57	4	2.89			
Total variation	9527.91	16				
*R* ^2^					0.9929	
*R* ^2^_Adj_					0.9837	
*R* ^2^_pre_					0.9037	
Adeq Precision					29.1717	
C.V. %					5.09	

Note: *p* ≤ 0.01 indicates that the effect of the factor on the composite score is highly significant (**).

**Table 3:** Response surface test factor level table.

Variant	Level of Factors			
(Factors)	Unit	−1	0	1
A Addition of sodium alginate	g	0.8	1	1.2
B Whey protein isolate addition	g	6	6.5	7
C Water-bath heating time	min	10	12	14

0, 1, −1 denote the center point value, maximum point value, and minimum point value in the response surface experiment.

## Data Availability

The original contributions presented in the study are included in the article, further inquiries can be directed to the corresponding author.
